# Supplementary material for: Iterative improvement in the automatic modular design of robot swarms
Source: PeerJ Comput Sci. 2020 Dec 7;6:e322. doi: 10.7717/peerj-cs.322 (PMC7924708; doi:10.7717/peerj-cs.322)
Supplement: Supplemental Information 3 [file peerj-cs-06-322-s003.zip › argos3/doc/api/standalone/a00523_source.html]

ARGoS: plugins/robots/generic/simulator/differential\_steering\_default\_actuator.h Source File


- Main Page
- Related Pages
- Namespaces
- Classes
- Files

- File List
- File Members

# plugins/robots/generic/simulator/differential\_steering\_default\_actuator.h

Go to the documentation of this file.

```
00001 
00007 #ifndef DIFFERENTIAL_STEERING_ACTUATOR_DEFAULT_H
00008 #define DIFFERENTIAL_STEERING_ACTUATOR_DEFAULT_H
00009 
00010 #include <string>
00011 #include <map>
00012 
00013 namespace argos {
00014    class CDifferentialSteeringDefaultActuator;
00015 }
00016 
00017 #include <argos3/plugins/robots/generic/control_interface/ci_differential_steering_actuator.h>
00018 #include <argos3/core/simulator/actuator.h>
00019 #include <argos3/plugins/simulator/entities/wheeled_entity.h>
00020 #include <argos3/core/simulator/entity/composable_entity.h>
00021 #include <argos3/core/utility/math/rng.h>
00022 
00023 namespace argos {
00024 
00025    class CDifferentialSteeringDefaultActuator : public CSimulatedActuator,
00026                                                 public CCI_DifferentialSteeringActuator {
00027 
00028    public:
00029 
00030       enum DIFFERENTIAL_STEERING {
00031          LEFT_WHEEL = 0,
00032          RIGHT_WHEEL = 1
00033       };
00034 
00035    public:
00036 
00040       CDifferentialSteeringDefaultActuator();
00041 
00045       virtual ~CDifferentialSteeringDefaultActuator() {}
00046 
00047       virtual void SetRobot(CComposableEntity& c_entity);
00048 
00049       virtual void Init(TConfigurationNode& t_tree);
00050 
00058       virtual void SetLinearVelocity(Real f_left_velocity,
00059                                      Real f_right_velocity);
00060 
00061       virtual void Update();
00062 
00063       virtual void Reset();
00064 
00065    protected:
00066 
00068       virtual void AddGaussianNoise();
00069 
00070    protected:
00071 
00072       CWheeledEntity* m_pcWheeledEntity;
00073       
00075       CRandom::CRNG* m_pcRNG;
00076       
00078       Real m_fNoiseStdDeviation;
00079 
00080    };
00081 
00082 }
00083 
00084 #endif
```

---

Generated on 10 Jul 2018 for ARGoS by 
 1.6.1
